# EAF2 Downregulation Recruits Tumor-associated Macrophages in Prostate Cancer through Upregulation of MIF

**DOI:** 10.1186/s12575-024-00247-0

**Published:** 2024-07-05

**Authors:** Tianyu Cao, Qian Sun, Xiaoqin Shi, Xiuke Lin, Qingyuan Lin, Jinchao Zhu, Junhao Xu, Di Cui, Youwei Shi, Yifeng Jing, Wenhuan Guo

**Affiliations:** 1 Department of Pathology, Shanghai Ninth People’s Hospital, Shanghai Jiaotong University School of Medicine, Shanghai, China; 2 Department of Urology, Shanghai General Hospital, Shanghai Jiaotong University School of Medicine, Shanghai, China; 3 Department of Urology, Tongren Hospital, Shanghai Jiaotong University School of Medicine, Shanghai, China; 4 Department of Pathology , Shanghai General Hospital, Shanghai Jiaotong University School of Medicine, Shanghai, China; 5 Fujian Provincial Key Laboratory of Innovative Drug Target Research, School of Pharmaceutical Sciences, Xiamen University, Xiamen, China; 6 Department of Urology, Chongqing University Three Gorges Hospital, Chongqing University, Chongqing, China

**Keywords:** Prostate cancer, EAF2, Tumor Associated Macrophage, MIF

## Abstract

**Background:**

The role of tumor inflammatory microenvironment in the advancement of cancer, particularly prostate cancer, is widely acknowledged. ELL-associated factor 2 (EAF2), a tumor suppressor that has been identified in the prostate, is often downregulated in prostate cancer. Earlier investigations have shown that mice with EAF2 gene knockout exhibited a substantial infiltration of inflammatory cells into the prostatic stroma.

**Methods:**

A cohort comprising 38 patients who had been diagnosed with prostate cancer and subsequently undergone radical prostatectomy (RP) was selected. These patients were pathologically graded according to the Gleason scoring system and divided into two groups. The purpose of this selection was to investigate the potential correlation between EAF2 and CD163 using immunohistochemistry (IHC) staining. Additionally, in vitro experimentation was conducted to verify the relationship between EAF2 expression, macrophage migration and polarization.

**Results:**

Our study demonstrated that in specimens of human prostate cancer, the expression of EAF2 was notably downregulated, and this decrease was inversely associated with the number of CD163-positive macrophages that infiltrated the cancerous tissue. Cell co-culture experiments revealed that the chemotactic effect of tumor cells towards macrophages was intensified and that macrophages differentiated into tumor-associated macrophages (TAMs) when EAF2 was knocked out. Additionally, the application of cytokine protein microarray showed that the expression of chemokine macrophage migration inhibitory factor (MIF) increased after EAF2 knockout.

**Conclusions:**

Our findings suggested that EAF2 was involved in the infiltration of CD163-positive macrophages in prostate cancer via MIF.

**Graphical abstract:**

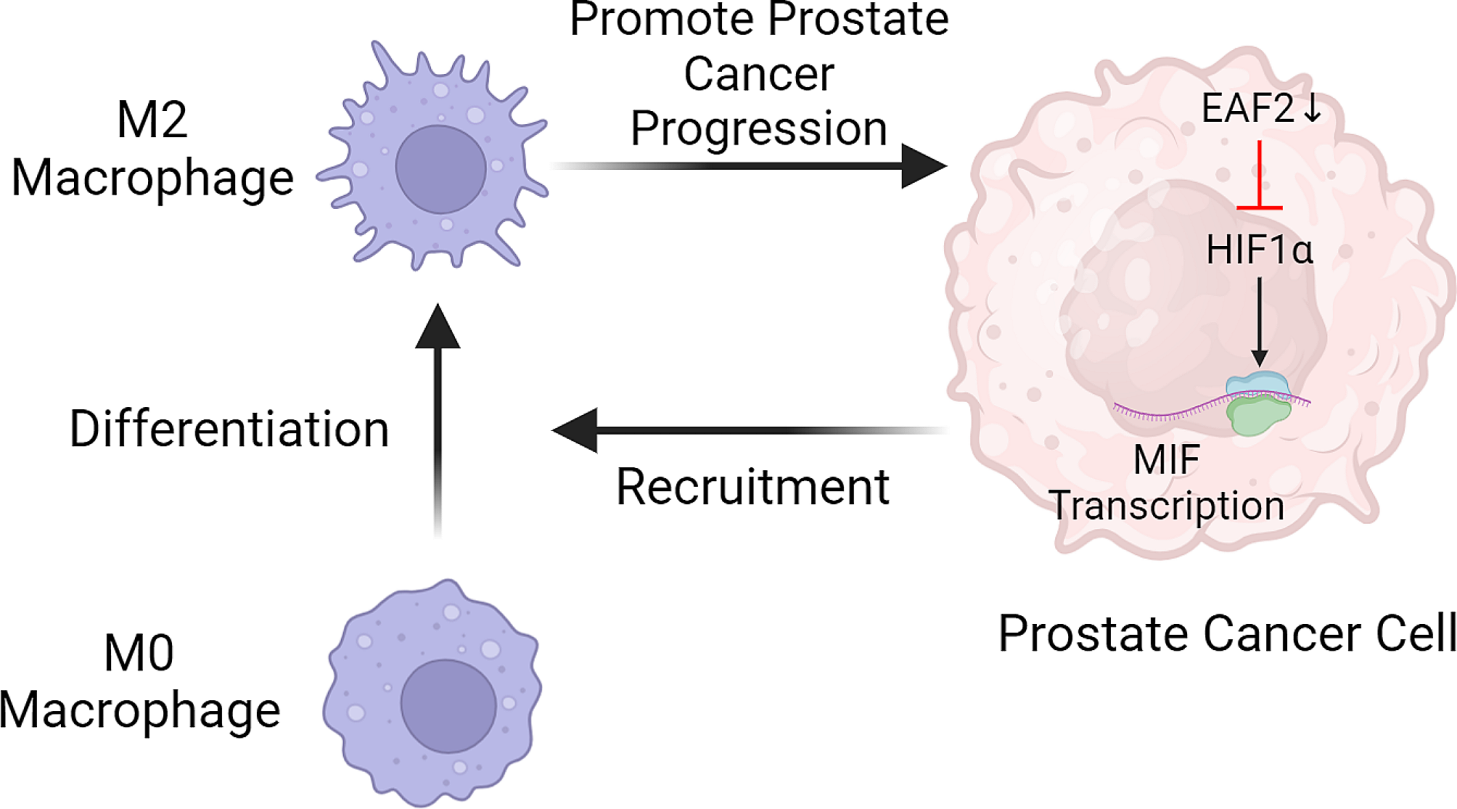

**Supplementary Information:**

The online version contains supplementary material available at 10.1186/s12575-024-00247-0.

## Highlights

EAF2 is associated with the progression of prostate cancer.

EAF2 is correlated with Gleason score of prostate cancer.

EAF2 is closely related to macrophages recruitment and polarition.

EAF2 recruits M2 macrophages through upregulation of MIF.

**Supplementary Information:**

The online version contains supplementary material available at 10.1186/s12575-024-00247-0.

## Introduction

Prostate cancer, the second most frequently diagnosed cancer in men, is also one of the top five causes of cancer-related mortality in men [1]. A significant proportion of prostate cancer patients are diagnosed in the advanced stages of the disease. For such patients, androgen deprivation therapy (ADT) is the standard treatment. However, despite initial responses to ADT, most patients eventually develop castration-resistant prostate cancer (CRPC) within 18–24 months, which has a poor prognosis [2]. Therefore, there is an urgent need to gain a better understanding of the mechanisms underlying cancer initiation and progression.

It is widely recognized that the tumor microenvironment, which includes a substantial population of macrophages, plays a significant role in cancer progression. Increasingly compelling evidence supports the idea that macrophages may contribute to cancer initiation and malignant progression. Macrophages can be broadly classified into classically activated (M1) and alternatively activated (M2) macrophages. In solid tumors, tumor-associated macrophages (TAMs) typically exhibit M2 phenotypes and display pro-malignancy activity. They have been strongly implicated in the progression, chemoresistance, and even checkpoint inhibitor (CPI) resistance of cancers [3–5]. In prostate cancer, TAMs have also been reported to play a pivotal role in cancer progression and are associated with resistance to ADT [6–9]. However, the mechanisms responsible for macrophage recruitment in prostate cancer remain elusive.

EAF2, also referred to as ELL-associated factor 2, has recently been identified as a tumor suppressor in prostate cancer and is frequently downregulated in this disease [10–14]. In recent years, several studies have been conducted to investigate the mechanisms underlying the tumor suppressive function of EAF2. EAF2 has been shown to regulate the activity of several signaling pathways through binding to different partners, including Smad3, HIF-1α, FOXA1, among others [15–17]. However, these studies have primarily focused on the direct role of EAF2 in cancer cells, and little is known about its effect on the tumor microenvironment. Moreover, a previous study has demonstrated intriguing findings that EAF2 knockout mice exhibited increased lymphatic dilation and chronic inflammation, including macrophage infiltration, as compared to wild-type controls [18]. These results suggested the possibility that EAF2 might play a role in mediating the recruitment of macrophages in prostate cancer, and further investigations are warranted to elucidate this potential mechanism.

This study aimed to examine the expression of EAF2 in prostate cancer and its relationship with the recruitment and polarization of TAMs. Our results indicated that the downregulation of EAF2 in prostate cancer may increase the accumulation of macrophages by promoting the production of migration inhibitory factor (MIF). Our findings suggest a previously unknown role for EAF2 as a tumor suppressor in prostate cancer.

## Materials and Methods

### Tissue Samples

We recruited 38 patients (with an age range of 45–81 years and a mean age of 69 years) who had recently been diagnosed with prostate cancer and underwent radical prostatectomy (RP) at Shanghai General Hospital. All patients were graded pathologically based on the Gleason scoring system and were subsequently divided into two groups: 21 patients with a Gleason score of ≤ 7 and 17 patients with a Gleason score of > 7. None of the patients had received any preoperative treatment for prostate cancer. Written informed consent was obtained from all patients, and the experimental protocol was approved by the Shanghai General Hospital of Shanghai Jiaotong University Medical School.

### Immunohistochemical Staining

Prostate cancer sections were deparaffinized, treated with heat-induced epitope retrieval (HIER) and incubated with primary antibodies against EAF2(1:200;Proteintech, CN), CD4 (original solution, Spincle, CN), CD8 (original solution, Gene Tech, CN), CD68 (original solution, Gene Tech, CN), CD20 (original solution, Gene Tech, CN), and CD163 (1:100; Dako, DK) for 1 h at room temperature. Then secondary antibody (GTvision, CN) was applied for 30 min at room temperature. The staining was developed using Diaminobenzidine and tissues were counterstained using hematoxylin then. The staining of samples was evaluated by two pathologists independently. The staining of EAF2 was divided into low expression group (EAF2-low) and high expression group (EAF2-high) according to the staining intensity compared with normal adjacent prostate. The staining intensity of the low expression group was weak and lower than that of normal adjacent prostate, and the high expression group was the same as or close to that of normal tissue. Count the number of CD163 positive staining cells in 5 individual cells using a blind method, and then calculate the average value.

### Cell Culture

LNCaP prostate cancer cells and human THP-1 cells were obtained from the Cell Bank of Type Culture Collection of the Chinese Academy of Sciences (Shanghai, CN) and maintained in RPMI 1640 medium supplemented with 10% FBS and 5% antibiotics. For stable EAF2 knockdown, LNCaP cells were incubated with LV-EAF2-RNAi or negative control (Genechem, CN) for 12 h and then treated with Puromycin(5ug/ml)for one week. For the conditioned media, the indicated cells were incubated with non-serum media for 48 h, and the culture media were collected and centrifuged for further experiments. For THP-1 cells differentiation, THP-1 cells were treated with phorbol myristate acetate (PMA, Sigma, USA) for 30 h to achieve macrophage differentiation(M0). Then M0 macrophages were cultured in the conditioned medium of LNCaP cells with or without EAF2 knockdown in petri dishes for 3 days to determine the effect of prostate cancer cells on the differentiation of M0 macrophages. Add 20ng/mL IL-4 (MCE, CN) and 20ng/mL IL-13 (MCE, CN) to M0 macrophages induced by THP-1 to induce their differentiation into M2 phenotype.

### Western Blot

Cells were lysed using RIPA buffer (NCM, China) with 1% protease inhibitor cocktail (Roche, IN). The concentration of protein was determined using the Pierce BCA Protein Assay (Thermo Scientific, USA). Total protein (20 µg) was boiled and separated on 10% SDS-PAGE gels and then transferred onto PVDF membranes (Millipore, USA). The membranes were blocked with skim milk and incubated with primary antibodies against EAF2 (1:1000, Proteintech, CN), MIF (1:1000, Proteintech, CN) at 4 °C overnight and then followed by secondary antibodies for two hours at room temperature. Protein bands were visualized using ECL (NCM, China) and exposed by the ECL Detection System (Thermo Scientific, USA).

### Preparation of RNA and Quantitative Real-time PCR

Total RNA was extracted from cells using TRIzol reagent (Invitrogen, USA) and the RNA reverse transcription was proceeded using a first-strand cDNA synthesis kit (Promega, USA). Real-time PCR was carried out using SYBR green mix (Thermo Scientific Waltham, USA). The sequences of primers used were presented below.

EAF2 forward: 5’-TTTGAAGTCATAGCGCACAGT-3’; EAF2 reverse: 5’-AATAGCGCAGCGGGATTCTC-3’; GAPDH forward: 5’-CGACCACTTTGTCAAGCTCA-3’, GAPDH reverse: 5’-AGGGGAGATTCAGTGTGGTG-3’; MIF forward: 5’-ATCGTAAACACCAACGTGCC − 3’, MIF reverse: 5’-TTGCTGTAGGAGCGGTTCTG-3’; TNF-α forward: 5’-CCTCTCTCTAATCAGCCCTCTG-3’, TNF-α reverse: 5’-GAGGACCTGGGAGTAGATGAG-3’; IL-6 forward: 5’-ACTCACCTCTTCAGAACGAATTG-3’, IL-6- reverse: 5’-CCATCTTTGGAAGGTTCAGGTTG-3’; CD68 forward: 5’-GGAAATGCCACGGTTCATCCA-3’, CD68 reverse: 5’-TGGGGTTCAGTACAGAGATGC-3’; CD 206 forward: 5’-TCCGGGTGCTGTTCTCCTA-3’, CD206 reverse: 5’-CCAGTCTGTTTTTGATGGCACT-3’; CD163 forward: 5’-TTTGTCAACTTGAGTCCCTTCAC-3’, CD163 reverse: 5’-TCCCGCTACACTTGTTTTCAC-3’; TGF-β forward: 5’-GGCCAGATCCTGTCCAAGC-3’, TGF-βreverse: 5’-GTGGGTTTCCACCATTAGCAC-3’.

### Cytokine Array and ELISA

Cytokines in the supernatant were measured using Human Cytokine Array Kit (R&D Systems, USA) according to the manufacturer’s instructions. Membranes were then scanned by a densitometer (Bio-Rad Laboratories, USA). Selected cytokines were further validated by ELISA using Quantikine ELISA Kit (R&D Systems, USA). The intensity was measured at 450 nm in a microplate reader (Thermo, USA).

### Migration Assays

Migration assays were performed in transwell inserts (8 µM, Corning, USA) placed in a 24-well plate. To test the chemotactic activity of tumor cells on M2 macrophages, M2 macrophages were seeded onto the top chamber and tumor-conditioned media with or without EAF2 knockdown were added into the bottom chamber. Migrated macrophages at the end of 24 h incubation were photographed and counted.

### Data Collection and Bioinformatic Analyses

The mRNA expression profiles and clinical data from prostate cancer patients from The Cancer Genome Atlas (TCGA) (https://portal.gdc.cancer.gov) database is collected. Transcripts per million reads (TPM) are used to standardise the HTSeq-FPKM Level 3 data. The survminer (version 0.4.9) and survival (version 3.2–10) R toolkit are used for plotting of survival curves. The GSVA (version 1.34.0) R toolkit is used for calculate the correlation between EAF2 expression and immune cells. The clusterProfiler (version 4.4.4) and GOplot (version 1.0.2) are used for the correlation calculation of gene enrichment analysis. We used the deseq2 package (version 1.36.0) in R language to test the difference between the two count matrices of the high expression group and the low expression group of EAF2, and screened out all the positive and negative genes. Then, based on the Spearman correlation coefficient and according to the following criteria: *p* < 0.05| Log2 ‑ FC |>1, and the differentially expressed genes were further analyzed. We used the org.hs.eg.db package to convert the ID of the input molecular list, and used the clusterprofiler (version 4.4.4) package to perform Kyoto Encyclopedia of genes and genes Gene Ontology (KEGG) and Gene Ontology (go) enrichment analysis, and calculated the zscore value corresponding to each enrichment entry through the goplot package (version 1.0.2). We also calculated the Pearson correlation between EAF2 and the marker genes of five immune pathways. All figures are drawn using the ggplot2 (version 3.3.6) package.

### Statistical Analysis

Data are presented as mean ± SEM. Differences between groups were analyzed with Student’s t-test or Chi-square test according to different sample types. Calculate the required sample size using the R package pwr (version 1.3-0). Statistical analyses were performed using SPSS 27.0 and Graphpad Prism 9.4. Differences were considered statistically significant when probability values<0.05 .

## Results

### Decreased Expression of EAF2 is Associated with Progression of Prostate Cancer

**Fig. 1:**
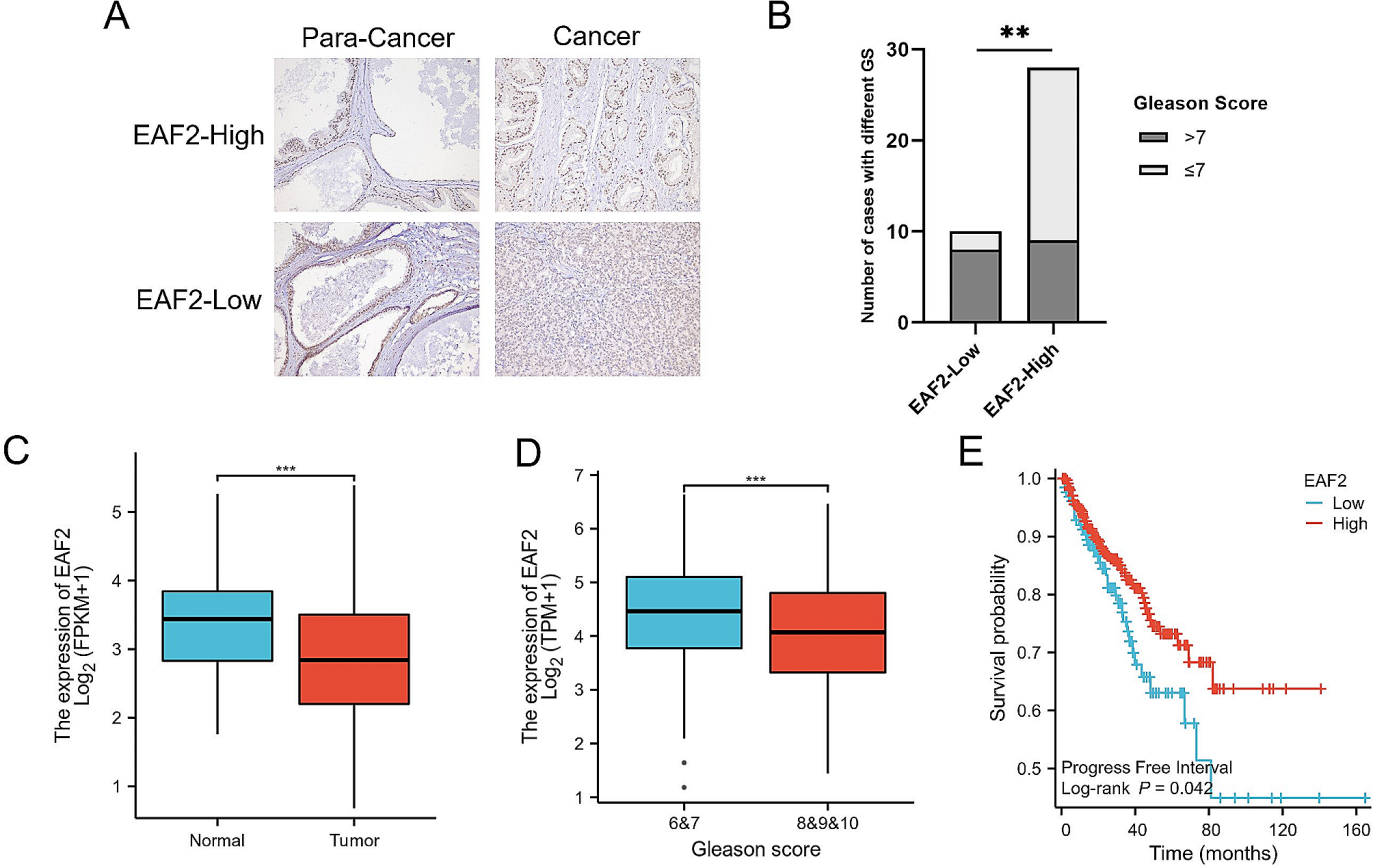
The expression of EAF2 in prostate cancer tissue. (**A**) Representative pictures of IHC staining of EAF2 in prostate cancers tissues and adjacent normal tissues with different expression of EAF2. (**B**) The proportion of cases with high GS and low GS in different EAF2 expression groups. (**C**) TCGA database analysis of expression levels of EAF2 in prostate cancers tissues compared with normal adjacent tissues. (**D**) TCGA database analysis of expression levels of EAF2 in prostate cancers tissues with different Gleason scores. (**E**) The PFI survival curves of patients in different EAF2 expression groups. EAF2 expression was negatively correlated with PFI. (** *p* ≤ 0.01; *** *p* ≤ 0.001)

To evaluate the expression of EAF2 in prostate cancer, we performed immunohistochemical staining against EAF2 on tissue samples from 38 prostate cancer patients (patient information is shown in Table 1). Based on the staining intensity of EAF2, we categorized the cases into two groups: EAF2 low expression (10 cases) and high expression groups (28 cases). According to the calculation of PWR package, each group needs at least 6 samples, so the sample size is sufficient. The low expression group of EAF2 was dominated by cases with a high Gleason score, whereas the high expression group had a higher proportion of cases with a low Gleason score (Gleason Score >7, 8/10 versus 9/28) (Fig. 1A, B). To corroborate our findings, we examined the TCGA database and found that tumor tissues exhibited lower EAF2 expression than normal tissues (Fig. 1C). Consistent with our results, EAF2 gene expression was reduced in the high Gleason score group compared to the low Gleason score group (Fig. 1D), and EAF2 expression was negatively correlated with Progress Free Interval (PFI) (Fig. 1E). These results suggest that downregulation of EAF2 expression, as a tumor suppressor, is associated with a higher tumor grade and poorer prognosis in prostate cancer. Notably, we observed that EAF2 was not downregulated in all tumor tissues (Fig. S1), indicating the possibility of organ-specific function of EAF2.

**Table 1:** Patient data

Patient ID	Age	EAF2 Expression Group	PSA	ISUP Score	TNM Stage	Positive margin	Extra-prostatic extension	Seminal vesicle invasion	Lymph Node Metastasis	Distant metastasis
1	74	Low	20.76	2	T2N1M0	Yes	No	No	Yes	No
2	75	Low	11.02	5	T3N0M0	Yes	Yes	Yes	No	No
3	66	Low	13.05	4	T2N0M0	No	No	No	No	No
4	78	Low	6.46	1	T2N0M0	No	No	No	No	No
5	75	Low	31.88	5	T2N0M0	No	No	No	No	No
6	77	Low	22.82	4	T3N0M0	No	Yes	Yes	No	No
7	63	Low	35.1	5	T3N1M0	Yes	Yes	No	Yes	No
8	66	Low	62.33	5	T2N1M1	Yes	No	No	Yes	Yes
9	78	Low	31.06	5	T3N0M0	Yes	Yes	No	No	No
10	75	Low	8.22	5	T3N0M0	No	Yes	No	No	No
11	69	High	14.32	5	T2N0M0	No	No	No	No	No
12	68	High	19.24	4	T3N0M0	No	Yes	No	No	No
13	70	High	8.54	2	T2N0M0	No	No	No	No	No
14	63	High	17.24	4	T2N0M0	No	No	No	No	No
15	74	High	8.77	3	T2N0M0	Yes	No	No	No	No
16	58	High	28.19	4	T2N0M0	No	No	No	No	No
17	68	High	6.9	2	T3N0M0	No	Yes	Yes	No	No
18	71	High	14.78	1	T2N0M0	Yes	No	No	No	No
19	61	High	7.92	2	T2N0M0	No	No	No	No	No
20	72	High	7.09	1	T2N0M0	No	No	No	No	No
21	45	High	8.7	2	T3N0M0	No	Yes	No	No	No
22	58	High	8.29	2	T3N0M0	Yes	No	No	No	No
23	63	High	29.46	2	T3N0M0	No	No	No	No	No
24	62	High	5.15	2	T3N0M0	Yes	No	No	No	No
25	77	High	37.89	1	T3N0M0	No	No	No	No	No
26	67	High	5.68	2	T3N0M0	Yes	Yes	Yes	No	No
27	66	High	13.43	4	T2N0M0	Yes	No	No	No	No
28	72	High	8.77	1	T2N0M0	No	No	No	No	No
29	80	High	8.57	1	T2N0M0	No	No	No	No	No
30	65	High	7.09	5	T2N0M0	Yes	No	No	No	No
31	74	High	11.93	3	T3N0M0	Yes	Yes	Yes	No	No
32	71	High	13.32	3	T2N0M0	Yes	No	No	No	No
33	62	High	6.71	3	T3N0M0	Yes	Yes	Yes	No	No
34	65	High	7.82	5	T3N0M0	Yes	Yes	No	No	No
35	72	High	5.25	3	T2N0M0	Yes	No	No	No	No
36	81	High	8.08	5	T3N0M0	Yes	Yes	No	No	No
37	75	High	6.27	3	T2N0M0	Yes	No	No	No	No
38	70	High	14.84	4	T2N0M0	No	No	No	No	No

### Decreased Expression of EAF2 Increases Number of Tumor-associated Macrophages in Prostate Cancer Specimens

**Fig. 2:**
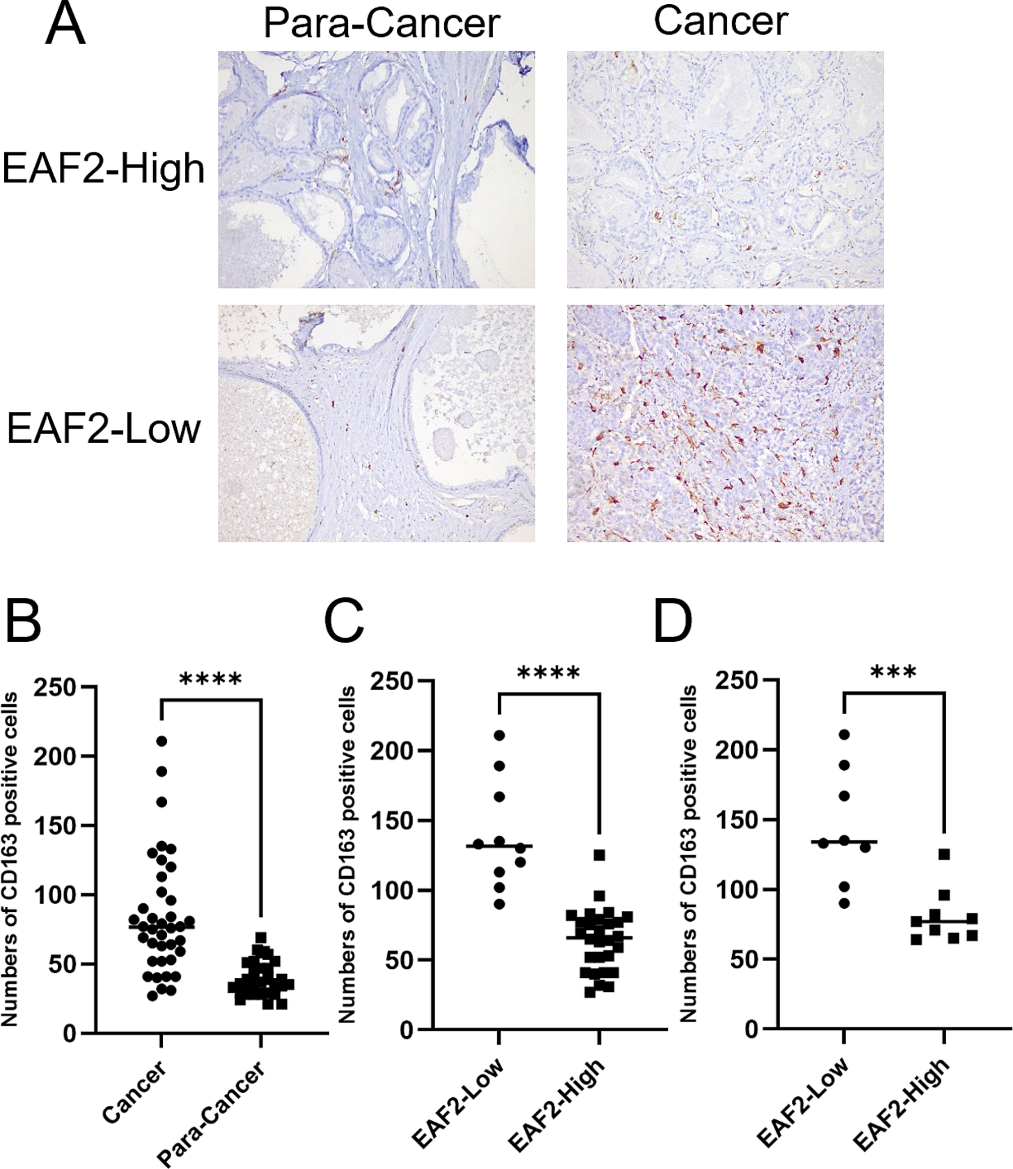
The expression of EAF2 in prostate cancer tissue. (**A**) Representative pictures of IHC staining of tumor associated macrophages using CD163 antibody in normal adjacent prostate and prostate cancers tissues. (**B**) Quantification and Statistical analysis of CD163 positive cells in normal adjacent prostate and cancer tissues. (**C**) Quantification and Statistical analysis of CD163 positive cells in EAF2-low and EAF2-high cancer tissues. (**D**) Quantification and Statistical analysis of CD163 positive cells in EAF2-low and EAF2-high cancer tissues with high GS. (*** *p* ≤ 0.001; **** *p* ≤ 0.0001)

To investigate the potential correlation between EAF2 expression and macrophage recruitment in prostate cancer patients, we conducted immunohistochemistry (IHC) staining against CD163. As demonstrated by an increase in the number of CD163 positive cells, there were more macrophages in cancerous tissues than in normal adjacent prostate tissues (Fig. 2A, B). Comparing the number of macrophages infiltrating cancer tissues with different levels of EAF2 expression, we found a significant increase in macrophages when EAF2 expression was low. This negative correlation between EAF2 expression and macrophage infiltration in prostate cancer tissues was observed (Fig. 2A, C). Since the low expression group of EAF2 had a higher proportion of high GS, and GS may also play a role in macrophage infiltration, we compared only cases with high GS in both groups and found a greater number of TAM infiltrations in the low EAF2 expression group than in the high EAF2 expression group (Fig. 2D). Furthermore, we analyzed the correlation between EAF2 expression and immune cell infiltration in the TCGA database and found a negative correlation between EAF2 and macrophage infiltration, although it did not reach statistical significance (Fig. S2A).

We found this trend in prostate cancer tissues by immunohistochemical staining (Fig. S2B). Additionally, EAF2 was negatively correlated with the expression of CD68 and CD163 (Fig. S3A). We also observed that high expression of CD163 was associated with a worse prognosis for patients (Fig. S3B). These results suggested that EAF2 expression might have a role in macrophage infiltration in prostate cancer.

### Knockdown of EAF2 Promotes M2 Macrophage Migration and Facilitates its Activation

**Fig. 3:**
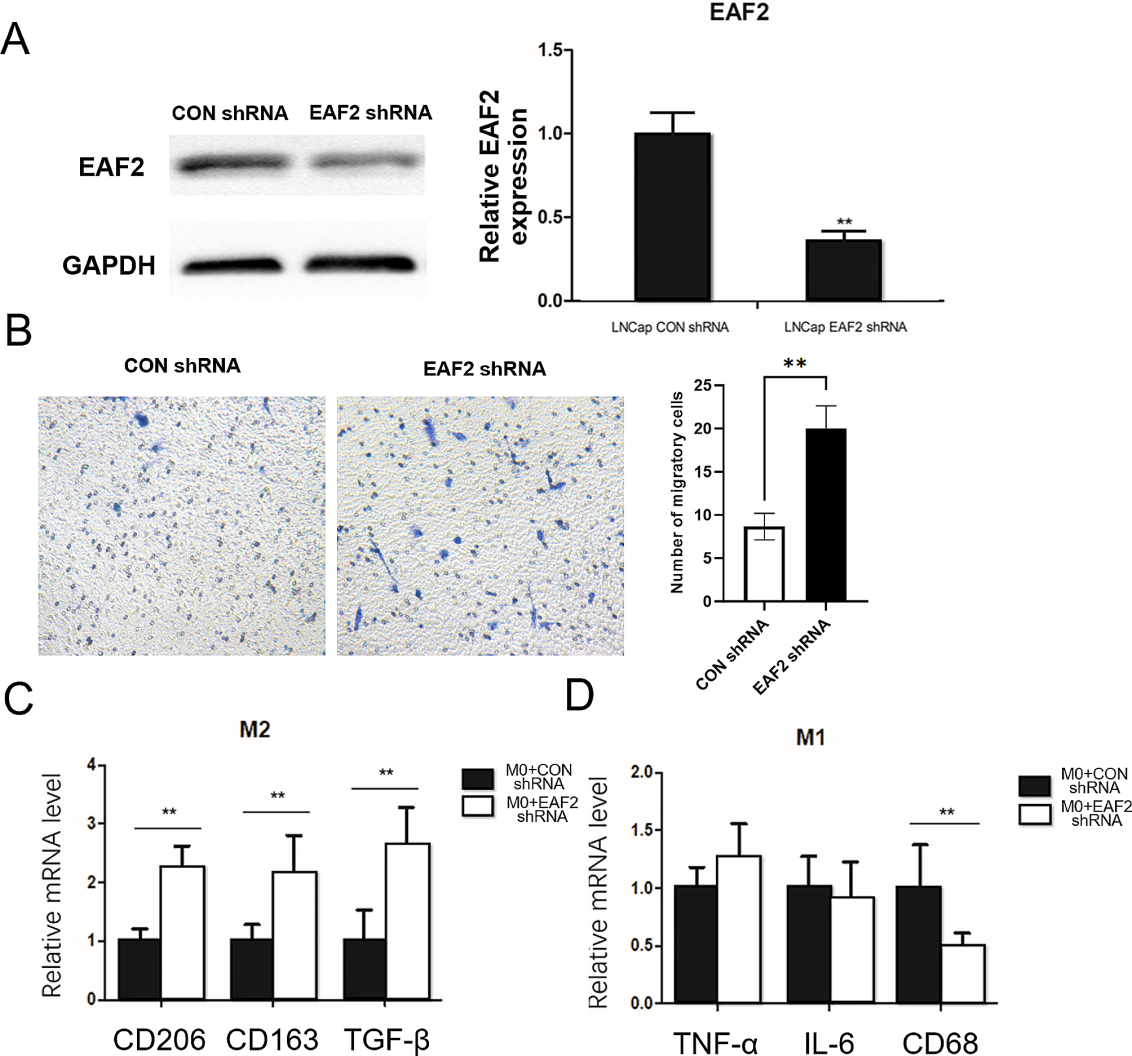
Down-regulation of EAF2 increased the migration of M2 macrophage and facilitated polarization of macrophages towards M2. (**A**) ShRNAs against EAF2 were stably transfected into LNCaP cells and the expression of EAF2 was detected using western blot and qPCR. (**B**) M2 macrophage cells were seeded onto the top chamber and conditioned media from LNCaP cells with or without EAF2 knockdown were added into the bottom chamber. After 24 h, M2 macrophage cells migrated into the bottom chamber were counted. **C-D.** M0 macrophages were co-cultured with LNCAP cells with or without EAF2 knocked down for 3 days, and the expression of M1 and M2 macrophage markers was examined by qRT-PCR. (** *p* ≤ 0.01)

To investigate the impact of EAF2 on macrophage recruitment, lentiviral constructs containing shRNAs targeting EAF2 were designed and transfected into LNCaP cells to stably knockdown EAF2 expression (Fig. 3A). We assessed whether the loss of EAF2 expression affects macrophage migration and polarization using these cells. Macrophage migration assays were performed using M2 macrophages with or without EAF2 knockdown LNCaP cell conditional media. Co-culture with EAF2 knockdown LNCaP cell conditional media resulted in a higher number of migrated M2 macrophages cells compared to co-culture with non-knockdown LNCaP cell conditional media (Fig. 3B). TAMs are typically skewed towards M2 polarization, and expression of M1 markers such as IL-6, TNF-α, and CD68, as well as M2 markers such as CD163, CD206, and TGF-β, are often used to identify TAM subtypes [19, 20]. To investigate whether the expression of EAF2 in LNCAP cells affects M0 macrophages to differentiate into M2 macrophages, a co-culture experiment was conducted, and qRT-PCR was performed to examine the expression of M1 and M2 macrophage markers. The results showed a significant increase in the mRNA expression of M2 macrophage markers after 72 h of co-culture with LNCAP cells in which EAF2 was silenced, compared to LNCaP CON shRNA (Fig. 3C). However, expression of M1 macrophage markers, including IL-6, TNF-α, and CD68, failed to increase after similar treatment, and CD68 expression was significantly suppressed (Fig. 3D). These findings suggest that EAF2 not only restrains the recruitment of macrophages but also suppresses their differentiation to the M2 phenotype.

### Knockdown of EAF2 Promotes the Secretion of MIF in LNCaP Cells

**Fig. 4:**
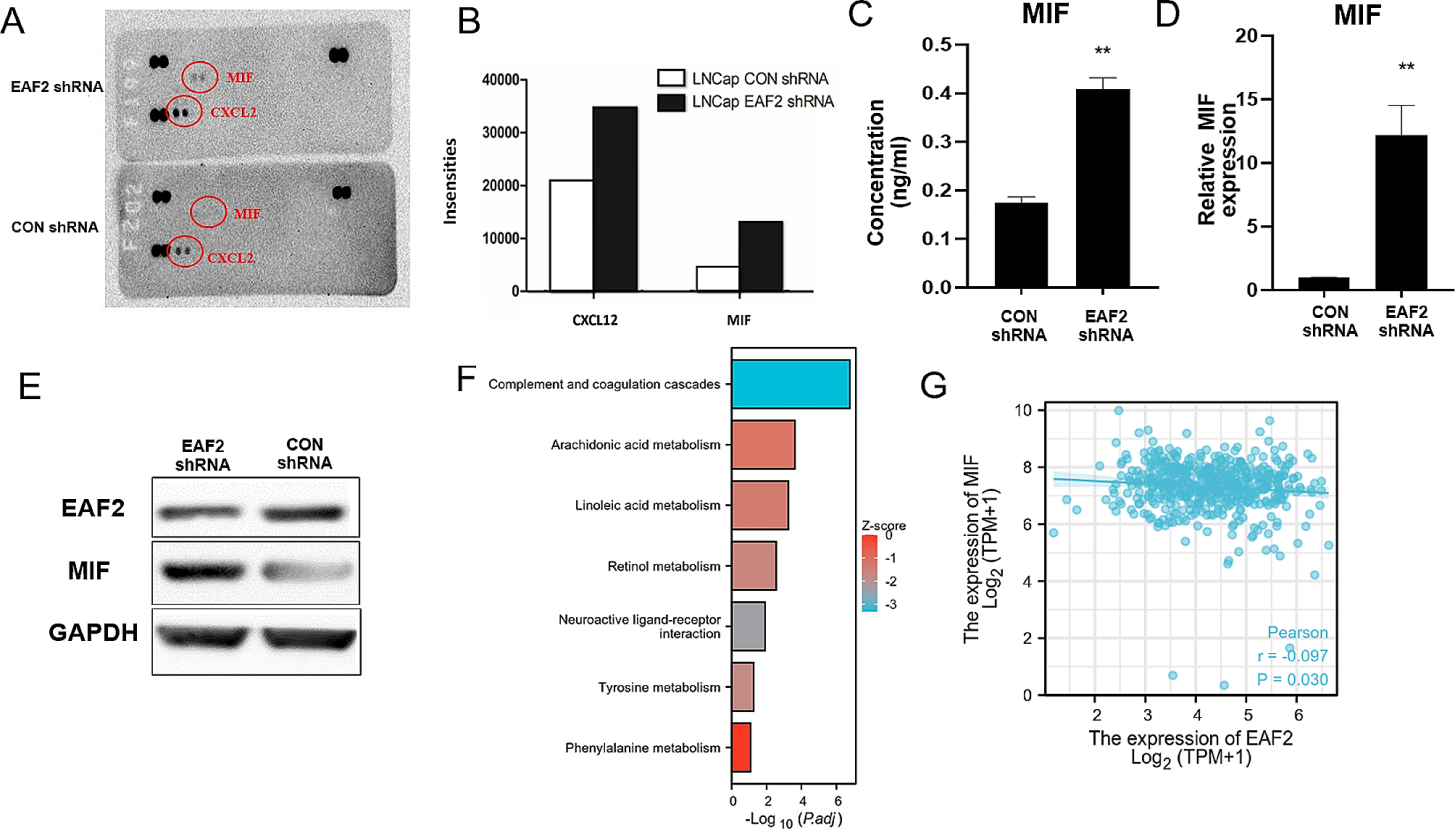
Knockdown of EAF2 in LNCaP cells increases the expression levels of MIF. (**A**) Conditioned media from LNCaP cells with or without EAF2 knockdown were analysed using Proteome Profiler Human Cytokine Array Panel A Kit. (**B**) Intensities of the blots were determined as pixel densities using Image J. (**C**) Conditioned media from LNCaP cells with or without EAF2 knockdown were examined for the concentrations of MIF by quantitative ELISA **D-E.** Protein and mRNA Expression levels of MIF in LNCaP cells with or without EAF2 knockdown were examined using qPCR and western blot. **F.** Kyoto Encyclopedia of Genes and Genomes (KEGG) pathway analysis results around EAF2.

In order to identify the potential inflammatory factors that may be involved in mediating the effect of EAF2 on M2 macrophages migration, we employed the Proteome Profiler Human Cytokine Array Panel A Kit to analyze the cytokine profiles in the supernatant from LNCaP cells with or without EAF2 knockdown. Our results showed that compared to the supernatant from LNCaP cells without EAF2 knockdown, the levels of cytokines MIF and CXCL12 were significantly higher in the supernatant secreted by LNCaP cells with EAF2 knockdown (Fig. 4A, B). Although CXCL12 was also found to be upregulated, the most pronounced change was observed for MIF, which is widely accepted to promote the chemotaxis of macrophages. Therefore, we focused our further investigation on MIF and validated its expression using ELISA assays (Fig. 4C). As anticipated, the secretion of MIF was observed to be upregulated in the cell culture supernatant with EAF2 knockdown. This finding was further confirmed by conducting qRT-PCR and Western blot analyses (Fig. 4D and E). To explore the association between EAF2 and MIF, we conducted Gene Set Enrichment Analysis (GSEA) using TCGA database. We first identified differentially expressed genes in prostate cancer patients with different EAF2 expression groups (Fig. S3C). Our results showed that the MIF-related metabolic pathways, including Tyrosine metabolic pathway and Phenylalanine metabolic pathway, were negatively correlated with EAF2 expression (Fig. 4F). We found that EAF2 was mainly positively correlated with immune activation related pathways (Fig. S3D). Based on this, we further analyzed the expression relationship between EAF2 and a variety of immune regulatory genes in prostate cancer, and drew the enrichment map of up-regulated gene set in prostate cancer (Fig. S3, S4). Additionally, we observed a direct negative correlation between the expression of EAF2 and MIF (Fig. 4G). These results suggest that downregulation of EAF2 can enhance the expression of MIF in LNCaP cells.

### Knockdown of EAF2 Promote the Migration of M2 Macrophages via MIF

**Fig. 5:**
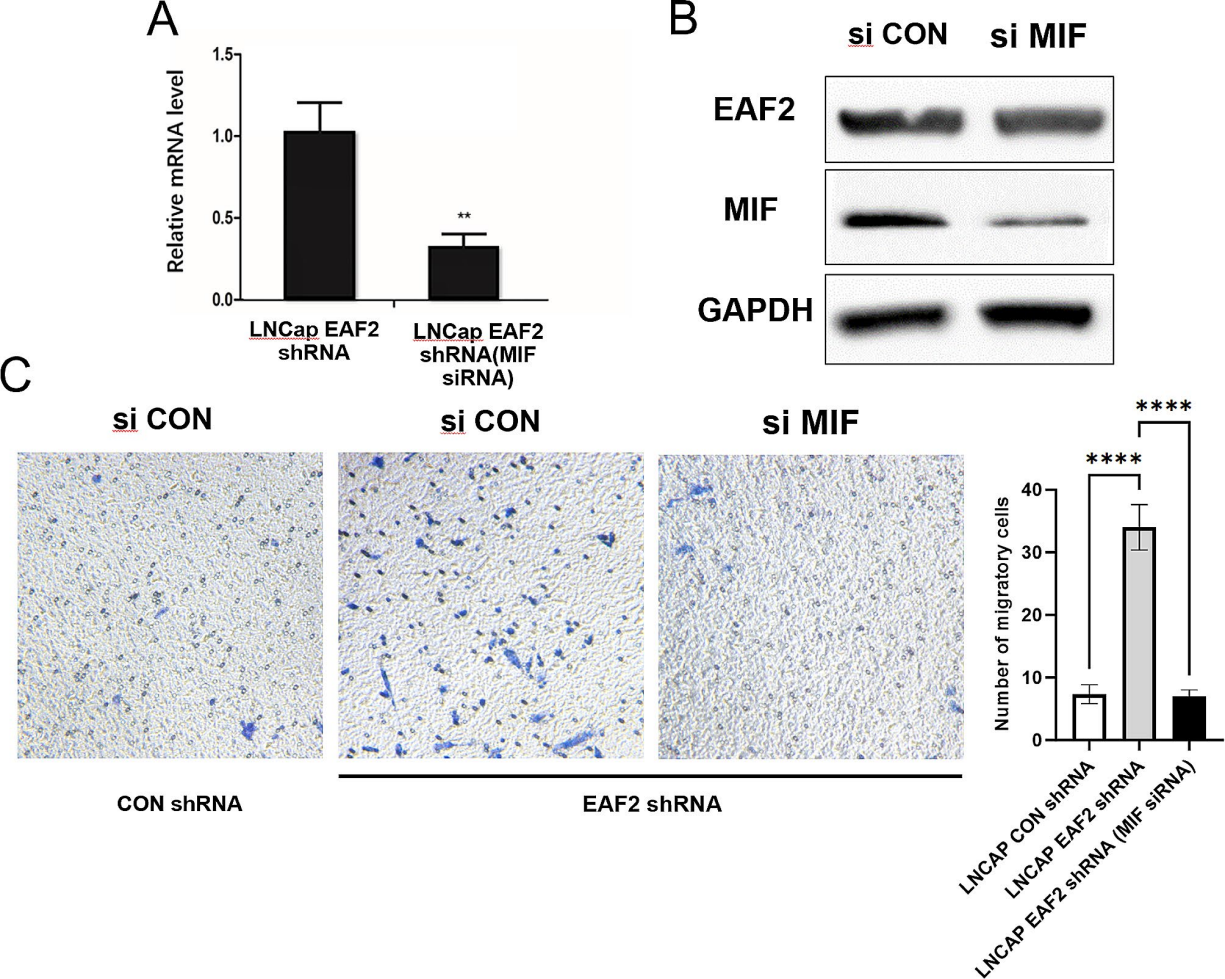
Knockdown of EAF2 in LNCaP cells promote the migration of M2 macrophage cells via MIF. (**A-B**) After EAF2 stably knocked down, LNCaP cells were transfected with siRNA targeting MIF, and protein and mRNA were extracted and subjected to qPCR and western blot to determine the expression levels MIF. (**C**) M2 macrophage cells were seeded onto the top chamber and co-cultured with conditioned media from LNCAP EAF2 shRNA cells transfected with siRNA targeted against MIF. After 24 h, M2 macrophage cells migrated into the bottom chamber were photographed and counted. (**** *p* ≤ 0.0001)

To investigate whether MIF is the critical factor involved in the recruitment function of EAF2 on M2 macrophages migration, siRNA targeting MIF was transfected into LNCAP EAF2 shRNA cells to knock down MIF expression. The effectiveness of knockdown was validated using qRT-PCR and western blot (Fig. 5A, B). Next, conditioned media were collected and co-cultured with M2 macrophage in migration assays to assess whether decreased EAF2 expression-induced increased migration of M2 macrophages could be blocked by knocking down MIF expression. The results showed that decreased MIF expression significantly reduced the migration of M2 macrophages, indicating that MIF is the critical cytokine through which LNCAP EAF2 shRNA cells inhibit the migration of M2 macrophages (Fig. 5C).

## Discussion

EAF2 has been widely studied as a tumor suppressor, with researchers striving to unravel the mechanisms underlying its tumor-inhibiting properties. However, there is limited literature on its potential association with the tumor microenvironment (TME), which accumulating evidence suggests plays a critical role in tumor behavior [21]. Our study is the first, to the best of our knowledge, to reveal that EAF2 can engage with the TME to facilitate the progression of prostate cancer. We used GSEA database to map EAF2 and immune related genes. This laid a foundation for further exploring the relationship between EAF2 and tumor immune microenvironment.

The tumor microenvironment consists of a heterogeneous group of cells, including immune cells, fibroblasts, pericytes, and endothelial cells [18]. TAMs are a critical component of the tumor microenvironment. In this study, we performed immunohistochemical staining on prostate cancer specimens using antibodies against EAF2 and CD163, a marker of M2 macrophages. Our findings demonstrate a significant correlation between EAF2 expression and the number of TAMs infiltrating tumor tissues.

Monocytes that are recruited from the peripheral blood serve as the primary source of TAMs. The recruitment of monocytes from the peripheral blood to the tumor microenvironment is the initial and critical step. After recruitment to tumors, monocytes differentiate into TAMs that can promote tumor progression and metastasis [19, 20]. We employed a co-culture system to investigate whether EAF2 could modulate the migration and differentiation of macrophages. As expected, knockdown of EAF2 led to increased migration of M2 macrophages. These results, combined with the immunohistochemical staining findings, suggest that EAF2 may play a role in monocyte attraction into the tumor microenvironment. This may also explain the findings from a previous study in mice [18].

Chemokines play a significant role in the progression of tumors by serving as a chemoattractant to induce immune cell infiltration to the TME, thus acting as a bridge between tumor cells and their microenvironment [19, 20]. Tumor cells are known to secrete diverse chemokines to facilitate the recruitment of inflammatory cells to infiltrate the tumor stroma [21, 22]. In our study, we employed the Proteome Profiler Human Cytokine Array to identify the possible chemokines that mediate the recruitment of macrophages by EAF2. Our findings indicated that MIF was one of the chemokines associated with EAF2 in the recruitment of macrophages.

MIF is a crucial cytokine involved in the pathogenesis of cancer and inflammatory diseases. Its role in promoting tumor progression and metastasis has been extensively studied, and its overexpression is often observed in various types of cancer [23–27]. MIF has also been shown to exhibit chemokine-like properties by modulating the recruitment of inflammatory cells through its receptor-mediated signaling pathways [28–32]. Notably, MIF has been reported to upregulate the expression and release of monocyte chemoattractant protein 1 (MCP1) via its interaction with CD74, which is believed to be a critical factor in the recruitment of monocytes and macrophages [33]. TAMs are recognized as essential components of prostate cancer progression [6, 7]. Therefore, it is hypothesized that EAF2 may not only have a direct effect on tumor cells but also an indirect effect through the recruitment of macrophages mediated by MIF, promoting prostate cancer progression. To examine the role of MIF in the recruitment of monocytes by EAF2, a co-culture system was used. Consistent with the hypothesis, knockdown of EAF2 in LNCaP cells increased M2 macrophages migration by upregulating MIF expression.

Several studies have investigated the regulation of MIF gene transcription and identified putative transcription factor binding sites in the MIF promoter. HIF1α has been reported to be a potent and rapid inducer of MIF expression. In contrast, EAF2 has been shown to suppress HIF1α transcriptional activity by disrupting its interaction with the coactivator CBP/p300 [15, 34]. This raises the possibility that EAF2 may regulate MIF expression through its interaction with HIF1α. However, further experiments are required to confirm this hypothesis.

In summary, our investigation offers novel insights into EAF2 as a potential tumor suppressor in prostate cancer. Our findings suggest that EAF2 may facilitate the accumulation of macrophages within prostate cancer tissue through MIF-mediated mechanisms. Furthermore, our results suggest a possible involvement of EAF2 in the differentiation of macrophages towards the M2 phenotype. Nonetheless, it is important to acknowledge that our research is still in its early stages, and further comprehensive studies are required to elucidate the underlying mechanisms and functional significance of these findings.

### Electronic Supplementary Material

Below is the link to the electronic supplementary material.

Supplementary Figure 1: Expression of EAF2 in various tumors and normal tissues. ACC, Adrenocortical carcinoma. BLCA, Bladder Urothelial Carcinoma. BRCA, Breast invasive carcinoma. CESC, Cervical squamous cell carcinoma and endocervical adenocarcinoma. CHOL, Cholangiocarcinoma. COAD, Colon adenocarcinoma. DLBC, Lymphoid Neoplasm Diffuse Large B-cell Lymphoma. ESCA, Esophageal carcinoma. GBM, Glioblastoma multiforme. HNSC, Head and Neck squamous cell carcinoma. KICH, Kidney Chromophobe. KIRC, Kidney renal clear cell carcinoma. KIRP, Kidney renal papillary cell carcinoma. LAML, Acute Myeloid Leukemia. LGG, Brain Lower Grade Glioma. LIHC, Liver hepatocellular carcinoma. LUAD, Lung adenocarcinoma. LUSC, Lung squamous cell carcinoma. MESO, Mesothelioma. OV, Ovarian serous cystadenocarcinoma. PAAD, Pancreatic adenocarcinoma. PCPG, Pheochromocytoma and Paraganglioma. PRAD, Prostate adenocarcinoma. READ, Rectum adenocarcinoma. SARC, Sarcoma. SKCM, Skin Cutaneous Melanoma. STAD, Stomach adenocarcinoma. TGCT, Testicular Germ Cell Tumors. THCA, Thyroid carcinoma. THYM, Thymoma. UCEC, Uterine Corpus Endometrial Carcinoma. UCS, Uterine Carcinosarcoma. UVM, Uveal Melanoma

Supplementary Figure 2: (**A**) Correlation between EAF2 expression and relative abundance of 24 types of immune cells. The size of dot corresponds to the absolute Spearman’s correlation coefficient values. (**B**) Representative pictures of IHC staining with CD4, CD8, CD20 and CD68 in prostate cancer

Supplementary Figure 3: (**A**) Correlations between CD163, CD68 and the expression of EAF2. (**B**) The PFI survival curves of patients in different CD163 expression groups. (**C**) Differentially expressed genes of EAF2 in prostate cancer patients (logFc>1 or <-1 and *p*-value<0.05). (**D**) Analysis of immune related pathways in prostate cancer patients with different EAF2 groups

Supplementary Figure 4: EAF2 is positively correlated with multiple immune regulatory genes in prostate cancer

Supplementary Figure 5: EAF2 is strongly and positively correlated with multiple immune checkpoint genes in prostate cancer

## Data Availability

The data that support the findings of this study are openly available in TCGA at https://www.cancer.gov/ccg/research/genome-sequencing/tcga.

## Abbreviations

EAF2: ELL associated factor 2
RP: Radical prostatectomy
IHC: Immunohistochemistry
TAMs: Tumor-associated macrophages
MIF: Migration inhibitory factor
ADT: Androgen deprivation therapy
CRPC: Castration-resistant prostate cancer
CPI: Checkpoint inhibitor
HIER: Heat-induced epitope retrieval
TCGA: The Cancer Genome Atlas
TPM: Transcripts per million reads
PFI: Progress Free Interval
GSEA: Gene Set Enrichment Analysis
TME: Tumor microenvironment
MCP1: Monocyte chemoattractant protein 1
