# Supplementary figures and images for: EAF2 Downregulation Recruits Tumor-associated Macrophages in Prostate Cancer through Upregulation of MIF
Source: Biol Proced Online. 2024 Jul 5;26:21. doi: 10.1186/s12575-024-00247-0 (PMC11225222; doi:10.1186/s12575-024-00247-0)

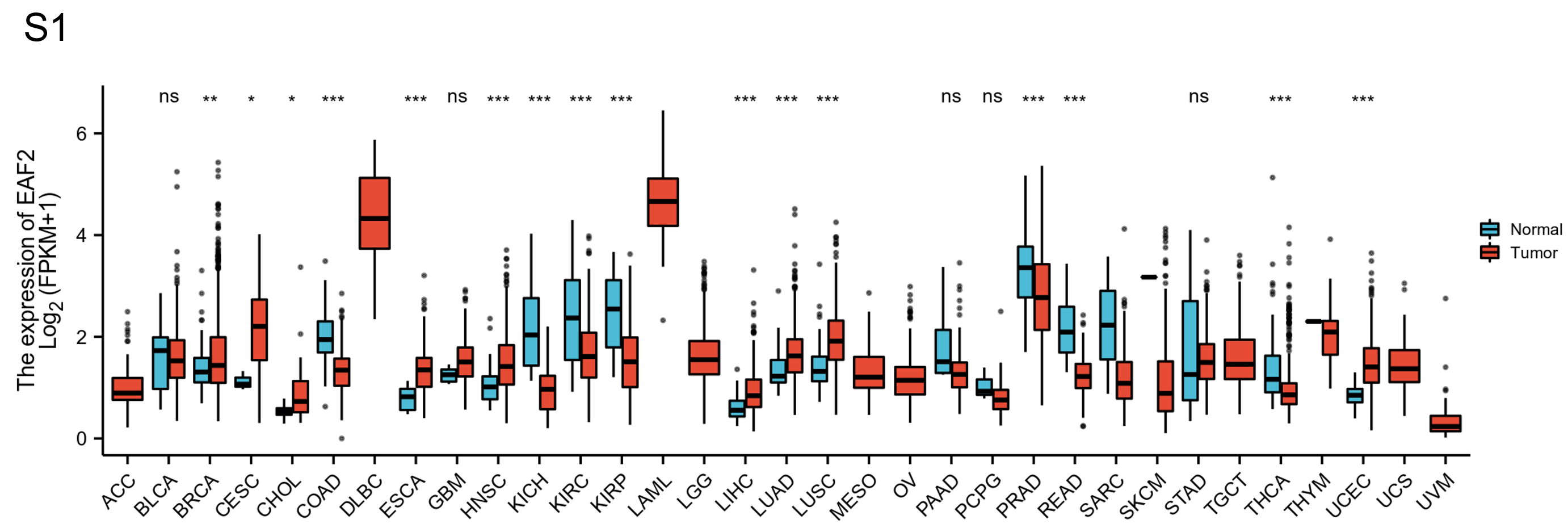

Supplement: Supplementary file 1 — Supplementary Figure 1: Expression of EAF2 in various tumors and normal tissues. ACC, Adrenocortical carcinoma. BLCA, Bladder Urothelial Carcinoma. BRCA, Breast invasive carcinoma. CESC, Cervical squamous cell carcinoma and endocervical adenocarcinoma. CHOL, Cholangiocarcinoma. COAD, Colon adenocarcinoma. DLBC, Lymphoid Neoplasm Diffuse Large B-cell Lymphoma. ESCA, Esophageal carcinoma. GBM, Glioblastoma multiforme. HNSC, Head and Neck squamous cell carcinoma. KICH, Kidney Chromophobe. KIRC, Kidney renal clear cell carcinoma. KIRP, Kidney renal papillary cell carcinoma. LAML, Acute Myeloid Leukemia. LGG, Brain Lower Grade Glioma. LIHC, Liver hepatocellular carcinoma. LUAD, Lung adenocarcinoma. LUSC, Lung squamous cell carcinoma. MESO, Mesothelioma. OV, Ovarian serous cystadenocarcinoma. PAAD, Pancreatic adenocarcinoma. PCPG, Pheochromocytoma and Paraganglioma. PRAD, Prostate adenocarcinoma. READ, Rectum adenocarcinoma. SARC, Sarcoma. SKCM, Skin Cutaneous Melanoma. STAD, Stomach adenocarcinoma. TGCT, Testicular Germ Cell Tumors. THCA, Thyroid carcinoma. THYM, Thymoma. UCEC, Uterine Corpus Endometrial Carcinoma. UCS, Uterine Carcinosarcoma. UVM, Uveal Melanoma [file 12575_2024_247_MOESM1_ESM.png]

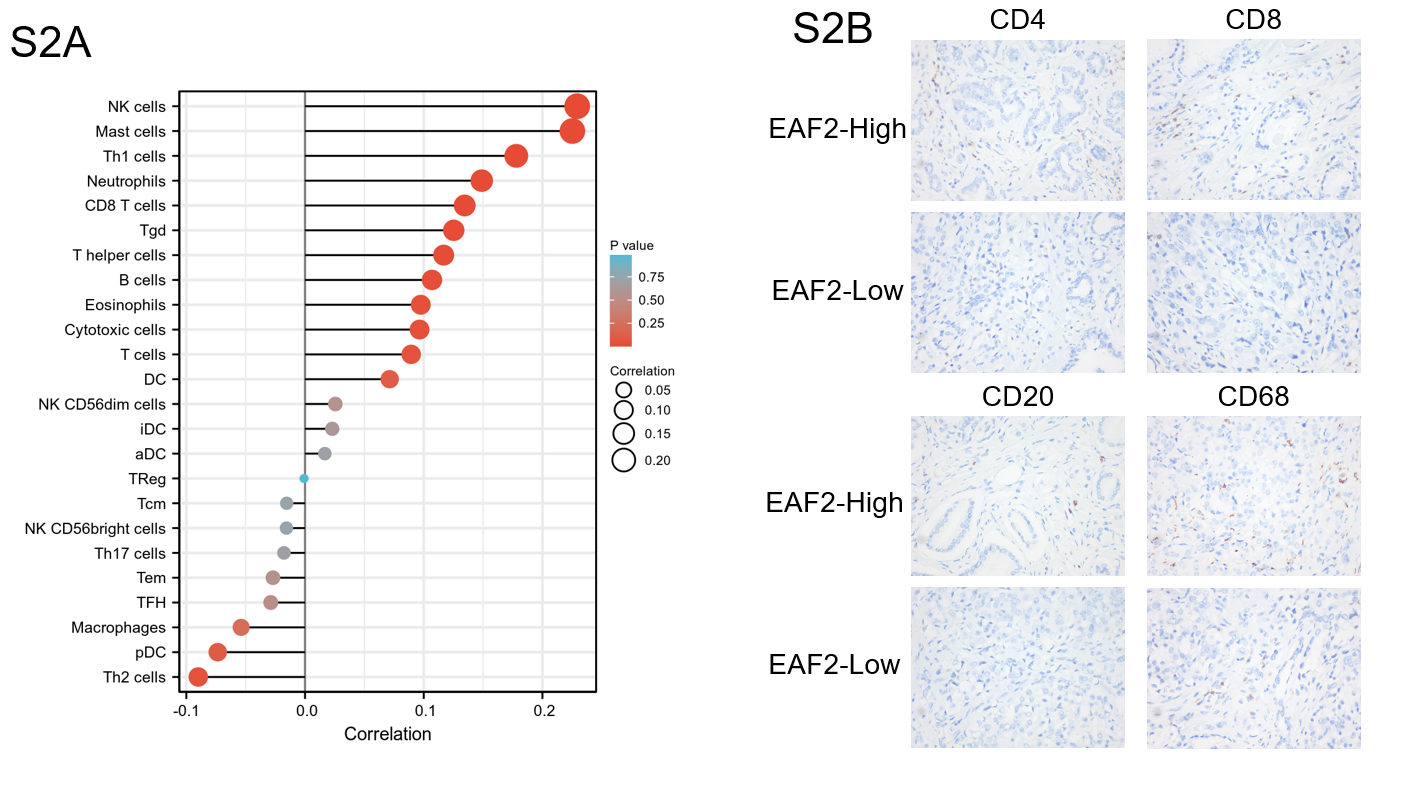

Supplement: Supplementary file 2 — Supplementary Figure 2: (A) Correlation between EAF2 expression and relative abundance of 24 types of immune cells. The size of dot corresponds to the absolute Spearman’s correlation coefficient values. (B) Representative pictures of IHC staining with CD4, CD8, CD20 and CD68 in prostate cancer [file 12575_2024_247_MOESM2_ESM.png]

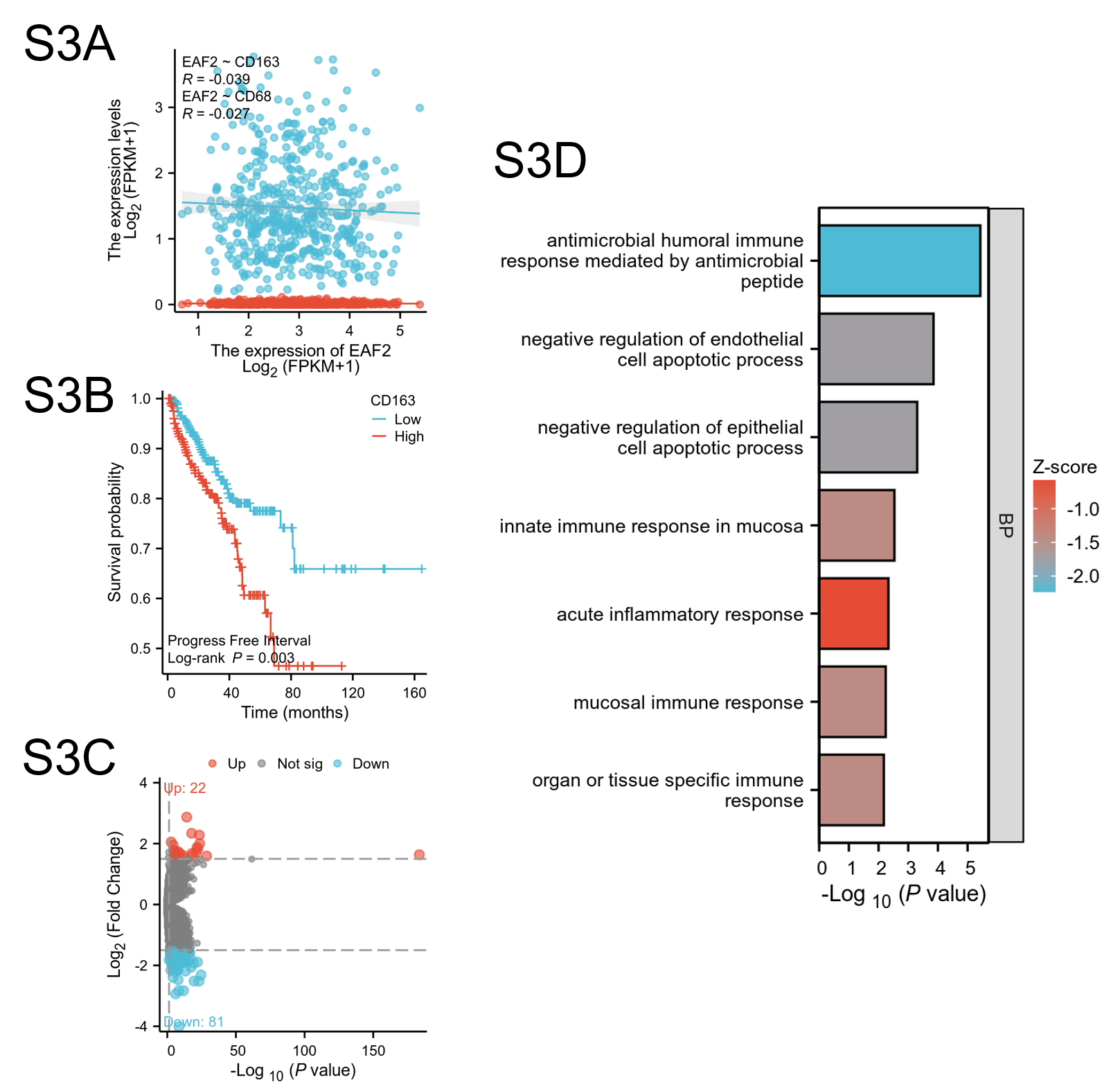

Supplement: Supplementary file 3 — Supplementary Figure 3: (A) Correlations between CD163, CD68 and the expression of EAF2. (B) The PFI survival curves of patients in different CD163 expression groups. (C) Differentially expressed genes of EAF2 in prostate cancer patients (logFc>1 or <-1 and p-value<0.05). (D) Analysis of immune related pathways in prostate cancer patients with different EAF2 groups [file 12575_2024_247_MOESM3_ESM.png]

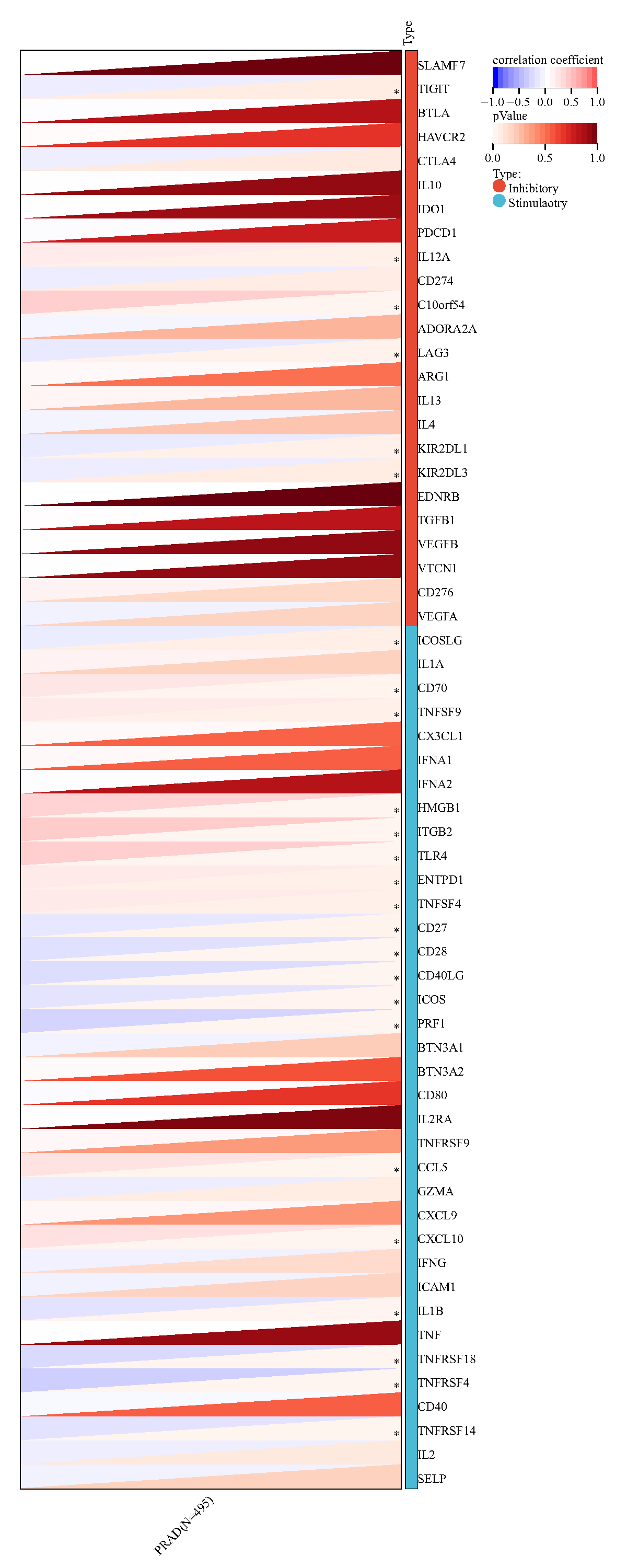

Supplement: Supplementary file 4 — Supplementary Figure 4: EAF2 is positively correlated with multiple immune regulatory genes in prostate cancer [file 12575_2024_247_MOESM4_ESM.png]

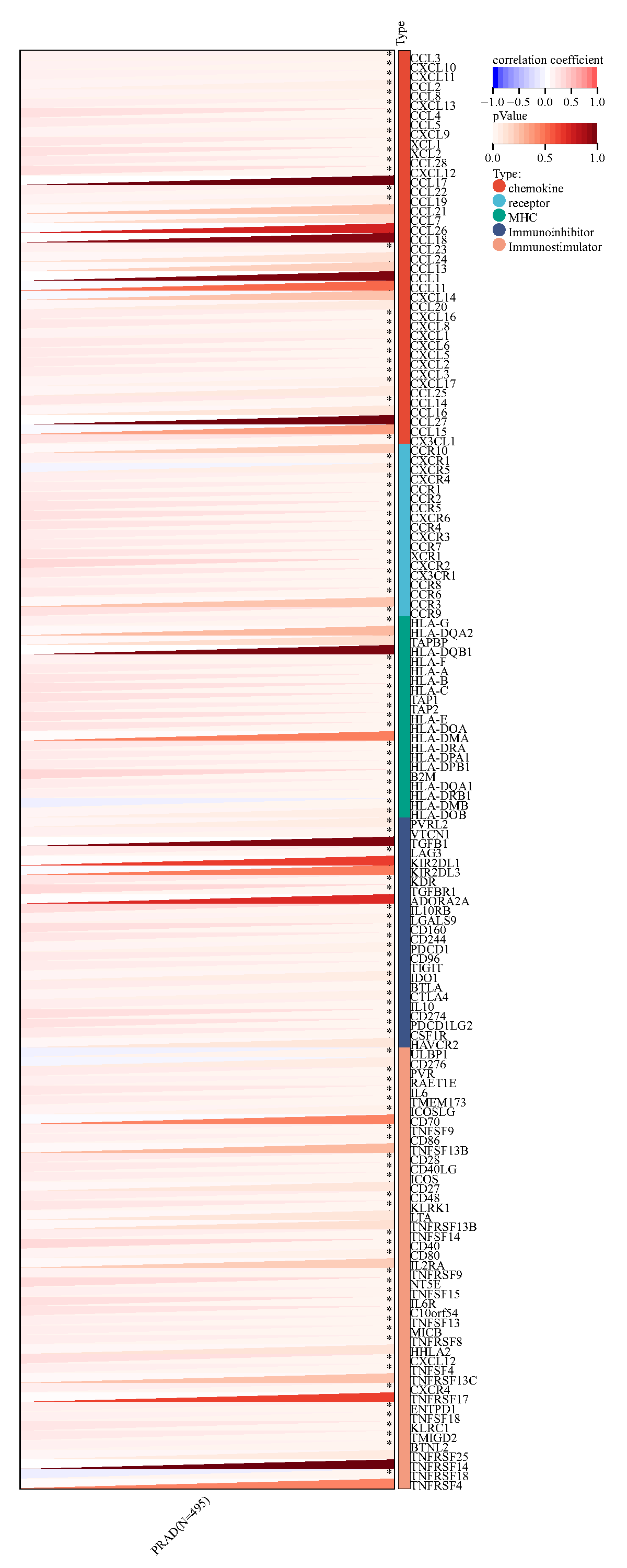

Supplement: Supplementary file 5 — Supplementary Figure 5: EAF2 is strongly and positively correlated with multiple immune checkpoint genes in prostate cancer [file 12575_2024_247_MOESM5_ESM.png]
